# End-of-life care in a COPD patient awaiting lung transplantation: a case report

**DOI:** 10.1186/1472-684X-9-6

**Published:** 2010-04-28

**Authors:** Daisy JA Janssen, Martijn A Spruit, Joan D Does, Jos MGA Schols, Emiel FM Wouters

**Affiliations:** 1 Program Development Centre, Ciro, centre of expertise for chronic organ failure, Horn, the Netherlands; 2 CAPHRI, Maastricht University, Maastricht, the Netherlands; 3 Proteion Thuis, Horn, the Netherlands; 4 Department of General Practice, Nursing home medicine, Faculty of Health Medicine and Life sciences/CAPHRI, Maastricht University, Maastricht, the Netherlands; 5 Department of Respiratory Medicine, Maastricht University Medical Centre + (MUMC+), Maastricht, the Netherlands

## Abstract

COPD is nowadays the main indication for lung transplantation. In appropriately selected patients with end stage COPD, lung transplantation may improve quality of life and prognosis of survival. However, patients with end stage COPD may die while waiting for lung transplantation. Palliative care is important to address the needs of patients with end stage COPD. This case report shows that in a patient with end stage COPD listed for lung transplantation offering palliative care and curative-restorative care concurrently may be problematic. If the requirements to remain a transplantation candidate need to be met, the possibilities for palliative care may be limited. Discussing the possibilities of palliative care and the patient's treatment preferences is necessary to prevent that end-of-life care needs of COPD patients dying while listed for lung transplantation are not optimally addressed. The patient's end-of-life care preferences may ask for a clear distinction between the period in which palliative and curative-restorative care are offered concurrently and the end-of-life care period. This may be necessary to allow a patient to spend the last stage of life according to his or her wishes, even when this implicates that lung transplantation is not possible anymore and the patient will die because of end stage COPD.

## Background

The prevalence and burden of Chronic Obstructive Pulmonary Disease (COPD) are expected to increase in the coming decades [1]. By 2020, COPD will rank third as cause of mortality and fifth as cause of disability worldwide [2].

The Global Strategy for the Diagnosis, Management, and Prevention of COPD describes the management of patients with COPD according to the severity of airflow limitation [1]. In patients with very severe COPD, surgical treatments like bullectomy, lung volume reduction surgery or lung transplantation need to be considered [1]. COPD is nowadays the main indication for lung transplantation [3]. In fact, in appropriately selected patients with end-stage COPD, lung transplantation may improve quality of life and prognosis of survival [4,5].

Referral for lung transplantation in COPD patients should only be considered in patients with progressive deterioration despite optimal treatment, including smoking cessation, maximal pharmacological treatment, pulmonary rehabilitation, long-term oxygen therapy and surgical treatment, if possible [4]. Listing for lung transplantation should occur when the expected prognosis of survival is significantly reduced, but exceeds the expected waiting time for lung transplantation [6]. Especially in COPD, where the survival benefit of lung transplantation remains debatable, quality of life needs to be taken into account, next to the survival benefit, in the decision making about listing for lung transplantation [4,6]. While lung transplantation may improve prognosis of survival and quality of life of some patients with end stage COPD, other COPD patients will die while waiting for a donor [7,8].

The importance of addressing the needs of patients with end stage COPD for palliative care (defined by the World Health Organization as *"an approach that improves the quality of life of patients and their families facing the problem associated with life-threatening illness, through the prevention and relief of suffering by means of early identification and impeccable assessment and treatment of pain and other problems, physical, psychosocial and spiritual" *[9]) has been shown before [10-12]. Recently has been described the need for a model of care in which comprehensive palliative care approaches are embedded within curative-restorative care [12,13]. However, goals of palliative care and curative-restorative care may be conflicting. For example, life-prolonging interventions may interfere with quality of life [14]. To date remains unknown whether and to what extent offering palliative care to patients with end stage COPD is influenced by being listed for lung transplantation.

The aim of this case report is to describe palliative and end-of-life care in a patient with end stage COPD listed for lung transplantation and to discuss the transition from curative-restorative care and palliative care to end-of-life care.

## Case Report

A 47-year old woman was admitted to our nursing home unit for patients with end stage lung disease because of COPD in June, 2006. She was admitted to the nursing home following hospitalisation because of an acute COPD exacerbation. The aim of admission to the specialized nursing home unit was providing nursing care and interdisciplinary care to improve her functional status.

She suffered from COPD since 1991. At time of admission to the nursing home her forced expiratory volume in the first second (FEV_1_) was 15% of predicted values. Her functional mobility, as assessed by the six minute walking distance (6MWD) was severely limited (155 meters; 23.6% of predicted values) [15]. In June 2006 she had a high body mass index (BMI, 27.6 kg/m^2^) and a low fat free mass index (14.4 kg/m^2^). Since 2001 she used long-term oxygen therapy. She participated in a comprehensive inpatient pulmonary rehabilitation program four times (2003, 2004, 2005 and 2006) at Ciro, centre of expertise for chronic organ failure in Horn, the Netherlands [16]. Since 2005 she was listed for lung transplantation. At that moment she needed two liters oxygen per minute for 24 hours a day. Her arterial carbon dioxide tension (PaCO_2_) was 6.2 kPa. Other surgical procedures, like lung volume reduction surgery were not possible because of homogeneous disease on High Resolution Computed Tomography (HRCT) [6].

Although she preferred to stay in her own home, it was not possible to deliver the care she needed in her home environment. Her husband suffered from chronic renal failure and underwent dialysis. He was waiting for kidney transplantation. In the nursing home she was offered an interdisciplinary patient-centred management program aimed at improving functional status to optimise the outcome of lung transplantation. Exercise training was the cornerstone of her management program and consisted of endurance training, resistance training of large muscle groups of upper and lower extremities, transcutaneous neuromuscular electrical stimulation of lower-limb muscles, unsupported arm exercises and inspiratory muscle training (IMT). The occupational therapist supported her with energy conservation and activity efficiency techniques, aimed at reducing her dependency in tasks of daily living.

Her disease trajectory was characterized by high sensation of dyspnea and frequent acute exacerbations. During her stay in the nursing home she deteriorated progressively. Her dyspnea increased (grade 5 on the Medical Research Council (MRC) dyspnea scale) [17]. She was suffering from clinically relevant symptoms of anxiety measured with the Hospital Anxiety and Depression Scale (HADS), a validated and reliable measurement instrument used widely in medically ill patients [18]. Therefore, she was treated by the psychosocial team with psychological individual counselling and art therapy. The high sensation of dyspnea and frequent exacerbations made it difficult for her to adhere to her daily exercise training program. Indeed, endurance training and even interval training became very hard for her to accomplish.

Because of her deterioration she was accepted for the high-priority list for lung transplantation in June, 2007. At that moment her PaCO_2 _had increased to 7,6 kPa and she was prescribed non-invasive positive pressure ventilation (NPPV). Unfortunately, because of severe anxiety for NPPV continuation was not possible. She suffered form dyspnea at rest and the use of opioids was necessary to achieve an acceptable level of daily symptom burden [12,19]. In December 2007 she received a call for lung transplantation. Unfortunately transplantation was not possible, because of insufficient quality of the donor lungs.

In February 2008, she was admitted to the Intensive Care Unit (ICU) because of progressive respiratory failure. After discharge she needed increasing dosages opioids to treat her dyspnea. In May 2008 she was again admitted to the hospital because of a very severe acute exacerbation of her COPD. At discharge to the nursing home her functional status had deteriorated severely (6MWD 55 m; 8.8% of predicted values). Moreover, her BMI was 31.8 kg/m^2^, in part because of oedema. She was admitted to the transplant centre in June 2008 to evaluate if she was still an appropriate candidate for lung transplantation. Echocardiography showed normal left ventricular systolic function, decreased left ventricular diastolic function because of impaired relaxation and normal right ventricular function. She was temporarily de-listed because of her BMI. The possibility that she would not survive to transplantation was discussed with her and her husband. In addition, the possibilities of palliative care and her treatment preferences were discussed. According to her wishes, goal of the interdisciplinary patient-centred management program remained improving functional status. In July 2008 her BMI was 29.8 kg/m^2^, her functional mobility improved (6MWD 90 meters; 14.2% of predicted values) and she was again listed with the intention to transplant. However, a few weeks later she was admitted to the hospital because of a severe acute exacerbation. Her BMI increased and was above 30 kg/m^2^. At discharge in September 2008 she was bedbound and used high dosages of corticosteroids. In addition, her opioid use had increased during the last months to 40 mg morphine subcutaneously a day. She had developed diabetes mellitus. After evaluation by the transplant centre she was once again temporarily de-listed and the requirements for listing with the intention to transplant were: a BMI below 30 kg/m^2 ^and lowering her dosage of opioids. At that moment her life expectancy appeared to be severely limited. In fact, also her quality of life was severely impaired, mainly because of high daily symptom burden. Decreasing the dosage of opioids caused unbearable suffering. She could hardly stand the burden of exercise training and the adherence to her strict diet affected her quality of life even more. At that moment her nursing home physician discussed again with her the expected poor prognosis of survival, the things that were important in her life and the possibilities for palliative care. She decided not to undergo mechanical ventilation or cardiopulmonary resuscitation in case of progressive respiratory failure or cardiac arrest.

In October 2008, the patient and her husband realized that achieving the requirements for becoming re-listed was not possible. They decided that the small chance for surviving to transplantation would not outweigh the burden of continuing curative-restorative treatment. At that moment she could be offered optimal end-of-life care, aimed at reducing suffering and improving quality of dying. Her physical and psychosocial symptoms could be optimally addressed. In addition, pastoral care supported her with her existential questions concerning her disease and dying. The dosage of opioids was titrated to her needs. Treatments that were burdensome for her were stopped. In addition, her nursing home physician discussed with her and her husband the process of dying and what dying might be like. Although it was very difficult for her to accept her unpromising situation after so many years of fighting for lung transplantation, she gradually accepted her situation. She was finishing the things she wanted to in her life and started with saying goodbye. In December 2008, two months after discontinuation of curative-restorative care, two-and-a-half year after admission to the nursing home and 3,5 years after listing for lung transplantation, she died quietly in the nursing home in the presence of her loved ones.

## Discussion

### Key findings

This case report describes the difficult process of decision making in a patient with end stage COPD awaiting lung transplantation and highlights the difficulty in balancing the needs for curative-restorative care to prolong life and the needs for palliative care to improve quality of life and quality of dying. The burden from curative-restorative care became too much for this patient towards the end of life. Therefore, it was necessary to offer end-of-life care aimed at improving quality of dying instead of curative-restorative care aimed at achieving again the requirements for listing for lung transplantation.

Since this patient was listed for lung transplantation she showed a progressive decline. The transplant criteria were regularly re-visited and several times she was temporarily de-listed, which had significant consequences for the patient and her loved ones. Nevertheless, the persistent scarcity of donor lungs asks for a careful selection of lung transplantation candidates [7]. Although the success rate of lung transplantation has gradually improved, morbidity and mortality rate immediately post-transplant remain considerably high [7]. Minimizing the chance of dying while on the waiting list is justified from the equity perspective, but wasting of donor lungs need to be avoided by de-listing of patients without a significant chance for successful transplantation [7,20].

Despite the fact that palliative care was gradually offered to this patient during the course of her disease, offering optimal end-of-life care was not possible if curative-restorative care had to be continued. For instance, it would not have been possible to titrate the dosage of opioids to her needs and burdensome treatments would have been continued. When it was clear that dying was unavoidable, the patient and her loved ones were prepared for her dying. This enabled them to finish the things they wanted to do and say goodbye.

Previously, differences in care have been shown at the end of life between patients with end stage lung disease listed for lung transplantation and patients not listed for lung transplantation. Indeed, cystic fibrosis (CF) patients who were listed for or underwent lung transplantation were more likely to die in the ICU than CF patients who were not listed for lung transplantation [21]. Moreover, CF patients who were listed for or received lung transplantation were more likely to be mechanically ventilated at or shortly before time of death. They were less likely to participate in discussions about end-of-life care than CF patients not listed for transplantation [22].

COPD patients may be confronted with acute life-threatening exacerbations. Therefore, timely advance care planning before such a crisis occurs, is necessary to guide treatment at the end of life [23]. Most patients with moderate to severe COPD have not discussed their preferences for end-of-life care with their treating physician [24,25]. However, discussing treatment preferences of patients with a life-limiting disease is necessary for the provision of optimal end-of-life care [26]. At time of referral for lung transplantation, life expectancy is greatly reduced and therefore the possibilities of palliative care and end-of-life care preferences should be discussed with the patient and loved ones. However, to date remains unknown whether and to what extent palliative care is discussed in COPD patients listed for lung transplantation.

Predicting prognosis of survival in COPD patients is a continuous challenge for the treating physician. The disease trajectory in COPD patients is marked typically by a gradually decline in health status and daily functioning and punctuated by acute exacerbations [27]. Every exacerbation can be life threatening and is associated with an increased risk of dying [27]. Because of this disease trajectory, the traditional dichotomous model of curative and palliative care in which curative care ends and palliative care starts at a certain moment is not appropriate in patients with COPD [12]. Therefore, the recent Official American Thoracic Society Clinical Policy Statement: Palliative Care for Patients with Respiratory Diseases and Critical Illnesses describes an individualized integrated model of palliative care in which palliative care starts when a patient suffering from a progressive respiratory disease becomes symptomatic [12]. Palliative care needs to be offered concurrent with curative-restorative care and the intensity of palliative care is titrated to the needs of the patient and the patient's family [12]. However, in patients listed for lung transplantation offering optimal palliative care may be limited when curative-restorative care aimed at staying in optimal physical condition for lung transplantation needs to be continued. Continuation of curative-restorative care may implicate that palliative care needs of COPD patients dying while listed for lung transplantation, are not optimally addressed. The patient's preferences for end-of-life care may ask for a clear distinction between the period in which palliative and curative-restorative care are offered concurrently and the end-of-life care period. This may be necessary to allow a patient to spend the last stage of life according to his or her wishes, even when this implicates that lung transplantation is not possible anymore and the patient will die because of end stage COPD. Choosing the right moment to discontinue curative care and offer only end-of-life care, and thereby accept that a patient becomes or stays de-listed and will die, is very difficult. Therefore, it is necessary to discuss the patient's preferences for end-of-life care early in the course of the disease and get to know and discuss what is important in the patient's life. These discussions can be framed as 'hope for the best and prepare for the worst' [10]. In this shared decision making process it is important to make careful analyses of the prognosis of survival, the chance for successful lung transplantation and the burden and benefits from curative-restorative care compared with the possibilities for palliative and end-of-life care. The decision to discontinue curative-restorative care and start end-of-life care aimed at relief of suffering and preparation for the unavoidable dying can only be made after careful discussions between the patient and his or her loved ones, the treating chest physician, the transplant centre and the palliative care physician.

Further research is necessary to elaborate the currently available knowledge concerning the needs for palliative care and the access to palliative care for COPD patients listed for lung transplantation. In addition, further research is necessary to study the preferences of COPD patients waiting for lung transplantation for care at the end of their life.

## Conclusions

This case report shows that in a patient with end stage COPD listed for lung transplantation offering palliative care and curative-restorative care concurrently may be problematic. The possibilities for palliative care may be limited if the requirements to remain a transplantation candidate need to be met. Discussing the possibilities of palliative care and the patient's treatment preferences is necessary to prevent that end-of-life care needs of COPD patients dying while listed for lung transplantation are not optimally addressed. The patient's end-of-life care preferences may ask for a clear distinction between the period in which palliative and curative-restorative care are offered concurrently and the period in which is offered only end-of-life care.

## Consent

Written informed consent was obtained from the patient's husband for publication of this case report. A copy of the written consent is available for review by the Editor-in-Chief of this journal.

## Competing interests

The authors declare that they have no competing interests.

## Authors' contributions

DJAJ was responsible for conception and design, acquisition of data and interpretation of data, drafting the manuscript and revising it critically for important intellectual content; MAS was responsible for conception and design, interpretation of data, drafting the manuscript and revising it critically for important intellectual content; JDD was responsible for acquisition of data, interpretation of data and revising the manuscript critically for important intellectual content; JMGAS was responsible for interpretation of data and revising the manuscript critically for important intellectual content; EFMW was responsible for interpretation of data and revising the manuscript critically for important intellectual content.

All authors have read and approved the final manuscript.

## Pre-publication history

The pre-publication history for this paper can be accessed here:

http://www.biomedcentral.com/1472-684X/9/6/prepub
